# Mean-Variance Portfolio Selection for Defined-Contribution Pension Funds with Stochastic Salary

**DOI:** 10.1155/2014/826125

**Published:** 2014-03-20

**Authors:** Chubing Zhang

**Affiliations:** School of Business, Tianjin University of Finance and Economics, Tianjin 300222, China

## Abstract

This paper focuses on a continuous-time dynamic mean-variance portfolio selection problem of defined-contribution pension funds with stochastic salary, whose risk comes from both financial market and nonfinancial market. By constructing a special Riccati equation as a continuous (actually a viscosity) solution to the HJB equation, we obtain an explicit closed form solution for the optimal investment portfolio as well as the efficient frontier.

## 1. Introduction

There are two radically different methods to design a pension fund: defined-benefit plan (hereafter DB) and defined-contribution plan (hereafter DC). In DB, the benefits are fixed in advance by the sponsor and the contributions are adjusted in order to maintain the fund in balance, where the associated financial risks are assumed by the sponsor agent; in DC, the contributions are fixed and the benefits depend on the returns on the assets of the fund, where the associated financial risks are borne by the beneficiary. Historically, DB has been more popular. However, in recent years, owing to the demographic evolution and the development of the equity markets, DC plays a crucial role in the social pension systems.

Because the member of DC has some freedom in choosing the investment allocation of their pension fund in the accumulation phase, they have to solve an optimal investment portfolio problem. Traditionally, the usual method to deal with it has been the maximization of expected utility of final wealth. Consistently with the economics and financial literature, the most widely used utility function exhibits constant relative risk aversion (CRRA), that is, the power or logarithmic utility function (e.g., [1–4]). Some papers use the utility function that exhibits constant absolute risk aversion (CARA), that is, the exponential utility function (e.g., [5]). Some papers also adopt the CRRA and CARA utility functions simultaneously (e.g., [6, 7]). However, Zhou and Li [8] think that in these situations the resulting portfolio policies are often shown to be myopic optimal policies, the utility functions from the investors are difficultly elicited, and trade-off information between the risk and the expected return is implicit which makes an investment decision much less intuitive. The objective of mean-variance can effectively solve the weaknesses of utility maximization for the optimal investment of DC. But there have been few pieces of literature on the mean-variance portfolio selection for DC pension funds. Thus, this paper studies the optimal investment portfolio selection of DC pension funds under the mean-variance model.

Markowitz [9] firstly designed the mean-variance portfolio selection model based on a trade-off between their expected return and risk, which is measured as the variance of the return. The most important contribution of this model is that it quantifies the risk by using the variance, which enables investors to seek the highest return after specifying their acceptable risk level. However, the mean-variance portfolio selection by Markowitz [9, 10] is only a single-period investment model. There have been continuing efforts in extending portfolio selection from the static single period model to dynamic multiperiod (e.g., [11–13]) or continuous-time models (e.g., [8, 14–16]). In the dynamic setting, Merton [17] made the successful and fundamental works by stochastic control methods, but the Merton model does not exactly fit the structure of mean-variance model. Recently, Zhou and Li [8] introduced a stochastic linear-quadratic (LQ) control framework to study the continuous-time version of the mean-variance problem without any constraints by the classical Riccati approach, where they derived closed-form strategy and obtained an explicit expression of the efficient frontier. After that, the stochastic LQ technique is widely used which is an effective method for mean-variance portfolio selection problems, for example, Li et al. [18], Chiu and Li [19], and Xie et al. [20]. This paper also adopts the stochastic LQ technique.

In addition, the optimal asset allocation for a pension fund involves quite a long period, generally from 20 to 40 years, so it follows that it becomes crucial to take into account the salary risk. Deelstra et al. [2] described the contribution flowed by a nonnegative, progressive measurable and square-integrable process and then studied optimal investment strategies for different examples of guarantees and contributions under the power utility. Battocchio and Menoncin [5] assumed that the stochastic salary's volatility came from both financial market and nonfinancial market and analyzed the behavior of the optimal portfolio with respect to salary under the exponential utility. Cairns et al. [3] considered the same assumption but used the member's final salary as a numeraire and then discussed various properties and characteristics of the optimal strategies with or without the presence of nonhedgeable salary risk under the power utility. Based on their researches, this paper also assumes that the stochastic salary risk comes from both financial market and nonfinancial market.

Our main objective in this paper is to find the optimal investment portfolio selection for DC pension funds with stochastic salary under the mean-variance model, which has not been reported in the existing literature. It is characterized by the following two aspects: (i) the optimal objective is not for the maximization of expected utility of final wealth but for the mean-variance of final wealth, so the decision-making process of weighing return and risk can be better understood; (ii) the stochastic salary's volatility contains both a hedgeable volatility whose risk source is the set of the financial market risk sources and a nonhedgeable whose risk source volatility does not the set of the financial market risk sources, so it is more suitable to the realistic economic background. We construct a special Riccati equation as a continuous (actually a viscosity) solution to the HJB equation and obtain an explicit closed form solution for the optimal investment portfolio as well as the efficient frontier.

## 2. Mathematical Model

In this section, we introduce the market structure and define the stochastic dynamics of the asset price and the salary.

We consider a complete and frictionless financial market which is continuously open over the fixed time interval [0, *T*], where *T* > 0 denotes the retirement time.

### 2.1. The Financial Market

Given an *n*-dimensional standard Brownian motion (*w*_1_,…,*w*_*n*_)^*T*^, we consider the complete probability space (*Ω*, *F*, *P*) generated by it; that is, to say, *F* is the filtration {*F*_*t*_}_*t*≥0_, with *F*_*t*_ = *σ* {*w*_1_(*s*),…, *w*_*n*_(*s*); 0 ≤ *s* ≤ *t*}.

The plan sponsor manages the fund in the planning interval [0, *T*] by means of a portfolio formed by *n* risky assets {*S* ^*i*^}_*i*=1_^*n*^, which are correlated geometric Brownian motions, generated by *w* = (*w*_1_,…,*w*_*n*_)^*T*^, and a riskless asset *S* ^0^, as proposed in Merton [17]; that is, whose evolutions are given by the following equations:

(1) dS0(t)=rS0(t)dt, S0(0)=1,dSi(t)=Si(t)(bidt+∑j=1nσijdwj(t)),Si(0)=si>0, i=1,2,…,n.

Here, *r* > 0 denotes the short risk-free rate of interest, *b*_*i*_ > 0 is the mean rate of return of the *i*th risky asset, and *σ*_*ij*_ is the covariance between asset *i* and *j*, for all *i*, *j* = 1,2,…, *n*. It is assumed that *b*_*i*_ > *r* for all *i*, so the sponsor has incentives to invest with risk.

### 2.2. The Stochastic Salary

We denote the salary at time *t* by *L*(*t*), which follows the stochastic differential equation (SDE):

(2) $$\begin{array}{c} dL\left(t\right)=\kappa L\left(t\right)dt+\eta L\left(t\right)dB\left(t\right),\;L\left(0\right)=L_{0}>0, \end{array}$$

where *κ* is an expected instantaneous growth rate of the salary and is a real constant. *η* is the instantaneous volatility of the salary, and both are constant parameters. Moreover, we assume that the instantaneous mean of salary is such that *κ* = *r* + *ν*, where *ν* is a real constant.

We suppose that there exists correlation *q*_*i*_ ∈ [−1,1] between *B* and *w*_*i*_, for *i* = 1,2,…, *n*. As a consequence, *B* is expressed in terms of {*w*_*i*_}_*i*=0_^*n*^ as $B(t)=\sqrt{1-q^{T}q}w_{0}(t)+q^{T}w(t)$ , where *q* ^*T*^ *q* ≤ 1 for *q* = (*q*_1_,*q*_2_,…,*q*_*n*_)^*T*^.

**Remark 1**

Equation (2) can be rewritten as $dL(t)=\kappa L(t)dt+\eta L(t)q^{T}w(t)+\eta L(t)\sqrt{1-q^{T}q}w_{0}(t)$ . *ηq* ^*T*^ is a volatility scale vector measuring how the risk source of stocks affects the salary, while $\eta \sqrt{1-q^{T}q}$ is a nonhedgeable volatility whose risk source does not the set of the financial market risk sources. This nonhedgeable risk source is represented by the one-dimensional standard Brownian motion *w*_0_(*t*) which is independent of *w*(*t*). In addition, both Battocchio and Menoncin [5] and Cairns et al. [3] assumed that the salary was affected by hedgeable and nonhedgeable risk sources. However, Battocchio and Menoncin [5] just studied a risky asset, while Cairns et al. [3] extended the investment opportunity set to *n* risky assets. Obviously, our assumption is similar to Cairns et al. [3].

### 2.3. The Wealth Process

According to the viewpoint of Cairns et al. [3], we consider that the contributions are continuous in the pension fund at the rate of *kL*(*t*).

Let *X*(*t*) denote the wealth of pension fund at time *t*. *π*_*i*_(*t*), *i* = 1,2,…, *n* represents the wealth amount invested in the *i*th risky asset at time *t*. We suppose that the investment strategy {Λ(*t*); *t* ≥ 0}, with Λ(*t*) = (*π*_1_(*t*),*π*_2_(*t*),…,*π*_*n*_(*t*))^*T*^, is a control process adapted to filtration {*F*_*t*_}_*t*≥0_, *F*-measurable, Markovian and stationary, stratifying *E*∫_0_^*T*^Λ(*t*)^*T*^Λ(*t*)*dt* < *∞*, where *E* is the expectation operator.

Therefore, the dynamics of the pension fund is given under the investment policy Λ by

(3) dX(t)=∑i=1nπi(t)dSi(t)Si(t)+(X(t)−∑i=1nπi(t))dS0(t)S0(t) +kL(t)dt, X(0)=X0,

where *X*_0_ stands for an initial wealth.

By taking into (1), the evolution of pension wealth can be rewritten as

(4) dX(t)=(rX(t)+∑i=1nπi(t)(bi−r)+kL(t))dt +∑i=1n ∑j=1nπi(t)σijdwj(t).

Next, we will assume the matrix notations *σ* = (*σ*_*ij*_), *b* = (*b*_1_,*b*_2_,…,*b*_*n*_)^*T*^, 1 = (1,1,…,1)^*T*^, and Σ = *σσ* ^*T*^. We take as given the existence of Σ^−1^, that is, to say, *σ* ^−1^. Finally the vector of standardized risk premia or Sharpe ratio of the portfolio is denoted by *θ* = *σ* ^−1^(*b* − *r*1).

So, we can rewrite (4) as

(5) dX(t)=(rX(t)+ΛT(t)(b−r1)+kL(t))dt +ΛT(t)σdw(t),

with the initial condition *X*(0) = *X*_0_.

For a prescribed target expected terminal wealth *EX*(*T*) = *K*, mean-variance portfolio optimization consists of determining a dynamic portfolio satisfying all the constraints of a given framework and minimizing the risk as measured by the variance of the terminal wealth, that is, minimizing

(6) $$\begin{array}{c} \mathrm{Var}X\left(T\right)=E{\left[X(T)-EX(T)\right]}^{2}=E{\left[X(T)-K\right]}^{2}. \end{array}$$

**Remark 2**

The investor expects a return above the risk free investment consisting of *π*_*i*_(*t*) = 0 for *i* = 1,2,…, *n* and whose associated wealth process *X*(·) satisfies

(7) $$\begin{array}{c} dX\left(t\right)=\left(rX\left(t\right)+kL\left(t\right)\right)dt,\;X\left(0\right)=X_{0},\;\;L\left(0\right)=L_{0} \end{array}$$

and has for solution *X*(*T*) = *X*_0_ *e* ^*rT*^ + ∫_0_^*T*^ *kL*(*t*)*e* ^*r*(*T*−*t*)^ *dt*, *L*(0) = *L*_0_.

This leads to the following natural assumption:

(8) $$\begin{array}{c} K\geq X_{0}e^{rT}+\int_{0}^{T}kL\left(t\right)e^{r(T-t)}dt,\;L\left(0\right)=L_{0}. \end{array}$$

## 3. The Optimal Control Problem

In this section, we provide the mean-variance model and then derive the Hamilton-Jacobi-Bellman (HJB) equation.

### 3.1. Mean-Variance Model

**Theorem 3**

A portfolio Λ(·) is said to be admissible if Λ(·) ∈ *L*_*F*_^2^ (0, *T*; *R* ^*n*^) for any *T* > 0 and the corresponding linear stochastic differential equations (2) and (5) have a unique solution pair (*X*(*t*), *L*(*t*)) corresponding to Λ(·). In this case, (*X*(·), *L*(*t*), Λ(·)) is called an admissible (wealth, salary, and portfolio) triple.

The following optimization problem parameterized by *K* ≥ *X*_0_ *e* ^*rT*^ + ∫_0_^*T*^ *kL*(*t*)*e* ^*r*(*T*−*t*)^ *dt*, *L*(0) = *L*_0_ is mean-variance portfolio selection:

(9)   min⁡    Var⁡X(T)=E[X(T)−K]2,subject  to EX(T)=K,(X(·),L(t),Λ(·))  is  admissible.

Problem (9) is called feasible if there exists at least one admissible pair satisfying *EX*(*T*) = *K*. Given *K*, the optimal strategy Λ*(·) of (9) is called an efficient strategy; this leads to a terminal wealth *X*(*T*) and the pair (*K*, Var⁡*X*(*T*)) is called an efficient point. The set of all efficient points, when the parameter *K* runs over [*X*_0_ *e* ^*rT*^ + ∫_0_^*T*^ *kL*(*t*)*e* ^*r*(*T*−*t*)^ *dt*, +*∞*), *L*(0) = *L*_0_, is called the efficient frontier.

Problem (9) is a dynamic quadratic convex optimization problem and hence has a unique solution. To find the optimal strategy corresponding to the constraint *EX*(*T*) = *K*, we introduce a Lagrange multiplier 2*μ* ∈ *R* (the factor 2 is introduced for computational convenience) and after rearranging terms there arrives at the new cost function

(10) J^(Λ(·),μ)=E[(X(T)−K)2+2μ(X(T)−K)]=E[X(T)−(K−μ)]2−μ2.

Letting *γ* = *K* − *μ* leads to the following optimal stochastic control problem (with the minimization over the set of strategies Λ(·)):

(11) min⁡    J¯(Λ(·),γ)=E[X(T)−γ]2−(K−γ)2,subject to (X(·),L(t),Λ(·))  being  admissible.

Note that, $\max_{\mu \in R}\min_{\Lambda (\cdot )}\hat{J}\left(\Lambda \left(\cdot \right),\mu \right)=\max_{\gamma \in R}\min_{\Lambda (\cdot )}\bar{J}\left(\Lambda \left(\cdot \right),\gamma \right)$ .

**Remark 4**

The link between problems (9) and (11) is provided by the Lagrange duality theorem; see, for example, Fu et al. [21]

(12) min⁡ Var⁡X(T)=max⁡μ∈R min⁡Λ(·)J^(Λ(·),μ)=max⁡γ∈R min⁡Λ(·)J¯(Λ(·),γ).

Obviously, for a fixed constant *γ*, problem (11) is clearly equivalent to

(13) min⁡    J(Λ(·),γ)=E[X(T)−γ]2,subject  to (X(·),L(t),Λ(·))  being  admissible.

### 3.2. The Optimization Program

By using the classical tools of stochastic optimal control, we define the value function

(14) H(t,X,L)=inf⁡Λ(·){(X(T)−γ)2 ∣ X(t)=x,L(t)=l;s.t.  (2),(5)},0<t<T.

The value function can be considered as a kind of utility function. Kramkov and Schachermayer [22] have demonstrated that the marginal utility of the value function is a constant. In addition, Jonsson and Sircar [23] have shown that the value function inherits the convexity of the utility function.

The maximum principle leads to the following Hamilton-Jacobi-Bellman (HJB) equation:

(15) Ht+inf⁡Λ(·){(rx+ΛT(t)(b−r1)+kl)Hx+κlHl+12η2l2Hll+12ΛT(t)ΣΛ(t)Hxx+ηlΛT(t)σqHxl}=0

with *H*(*T*, *x*, *l*) = (*x*−*γ*)^2^, where *H*_*t*_, *H*_*x*_, *H*_*l*_, *H*_*xx*_, *H*_*ll*_, and *H*_*xl*_ denote partial derivatives of first and second orders with respect to time, wealth, and salary.

The first order minimizing conditions for the optimal strategies Λ*(*t*) is

(16) $$\begin{array}{c} \Lambda^{*}\left(t\right)=-\Sigma^{-1}\left(b-r1\right)\frac{H_{x}}{H_{xx}}-\eta l\sigma^{-T}q\frac{H_{xl}}{H_{xx}}. \end{array}$$

Putting this in (15), we obtain a partial differential equation (PDE) for the value function *H*

(17) Ht+(rx+kl)Hx−12θTθHx2Hxx+κlHl+12η2l2Hll−ηlθTqHxHxlHxx−12η2l2qTqHxl2Hxx=0,

with *H*(*T*, *x*, *l*) = (*x*−*γ*)^2^.

## 4. Optimal Portfolio and Efficient Frontier

In this section, we find the optimal portfolio and the efficient frontier for the problems (9), (11), and (13).

### 4.1. Optimal Portfolio

We conjecture a solution to (17) with the following form:

(18) H(t,x,l)=β0(t)+β1(t)x+β2(t)l +β3(t)x2+β4(t)l2+β5(t)xl,

with the boundary conditions given by

(19) β0(T)=γ2,  β1(T)=−2γ,  β3(T)=1,β2(T)=β4(T)=β5(T)=0.

Therefore,

(20) $$\begin{array}{c} \Lambda^{*}\left(t\right)=-\Sigma^{-1}\left(b-r1\right)\left(x+\frac{\beta_{1}}{2\beta_{3}}+\frac{\beta_{5}}{2\beta_{3}}l\right)-\eta l\sigma^{-T}q\frac{\beta_{5}}{\beta_{3}}. \end{array}$$

The following ordinary differential equations are obtained for the above coefficients:

(21)  β˙0=14θTθβ12β3,  β0(T)=γ2, β˙1=(θTθ−r)β1,  β1(T)=−2γ,

(22)  β˙2=−κβl+(k+12θTθ)β1+12ηθTqβ1β5β3,  β2(T)=0, β˙3=(θTθ−2r)β3,  β3(T)=1, β4(T)=0, β˙5=−(2r+κ−θTθ−ηθTq)β5−2kβ3,  β5(T)=0.

(23)  β˙4=−(2κ+η2)β4+(θTθ+2ηθTq+η2qTq)β524β3−kβ5, β4(T)=0, β˙5=−(2r+κ−θTθ−ηθTq)β5−2kβ3,  β5(T)=0.

Obviously, the method of resolution of this system is standard. Equation (21) has, for solution, *β*_1_(*t*) = −2*γe* ^(*r*−*θ* ^*T*^ *θ*)(*T*−*t*)^; (22) also has, for solution, *β*_3_(*t*) = *e* ^(2*r*−*θ* ^*T*^ *θ*)(*T*−*t*)^.

Then, by substituting the expression for *β*_3_(*t*) into (23), we can obtain

(24) β5(t)=2kκ−ηθTq(e(2r−θTθ)(T−t)(e−(κ−ηθTq)t−1)−e−(κ−ηθTq)T+1).

Finally, by substituting *β*_1_(*t*), *β*_3_(*t*), and *β*_5_(*t*) into (20), the optimal investment strategy in the risky assets is given by

(25) $$\begin{array}{c} \Lambda^{*}\left(t\right)=-\Sigma^{-1}\left(b-r1\right)\left(x-\gamma +\frac{1}{2}f\left(t\right)l\right)-\eta l\sigma^{-T}qf\left(t\right), \end{array}$$

where

(26) f(t)=2kκ−ηθTq(e−(2r−θTθ)(T−t)(1−e−(κ−ηθTq)T)+e−(κ−ηθTq)t−1).

**Proposition 5**

With the above notation and for a given *γ*, *T* > 0 and

(27) $$\begin{array}{c} K\in \left[X_{0}e^{rT}+\int_{0}^{T}kL\left(t\right)e^{r(T-t)}dt,+\infty \right),\;L\left(0\right)=L_{0}, \end{array}$$

an optimal investment strategy corresponding to the problems (11) and (13) is

(28) Λ∗(t)=−Σ−1(b−r1)(X(t)−γ+12f(t)L(t)) −ησ−Tqf(t)L(t),

where,

(29) f(t)=2kκ−ηθTq(e−(2r−θTθ)(T−t)(1−e−(κ−ηθTq)T)+e−(κ−ηθTq)t−1).

**Remark 6**

The optimal portfolio in risky assets for the investor with mean-variance model can be divided into three parts.

The first part, −Σ^−1^(*b* − *r*1)*X*(*t*), is always negative and decreases with the wealth level, so we can denote it as a “pension wealth factor.” The higher the wealth level the investor has the lower the capital amount are invested in risky assets.

The second part is Σ^−1^(*b* − *r*1)*γ*. It is always positive, while the parameter *γ* depends on the equation *γ* = *K* − *μ*. So we can denote it as a “terminal wealth expectation factor,” which reflects the mean-variance intrinsic feature, that is, the weighing between mean and variance.

The third part, −(1/2Σ^−1^(*b* − *r*1) + *ησ* ^−*T*^ *q*)*f*(*t*)*L*(*t*), is mainly relative to the salary and reflects how the plan member's salary affects the optimal investment strategy. So we call it a “stochastic salary factor.” But we difficultly judge the sign of the “stochastic salary factor” for the several parameters' value that cannot be determined.

### 4.2. Efficient Frontier

In this section, we derive the efficient frontier for the portfolio selection problem (9); that is, we specify the relation between the variance and the expected value of the terminal wealth for every efficient strategy.

By substituting the optimal strategy (25) into the dynamics of the wealth equation (5), we obtain

(30) dX(t)=((r−θTθ)X(t)+kL(t)−θTθ(12f(t)L(t)−γ))dt −(θT(X(t)−γ+12f(t)L(t))+qTηL(t)f(t))dw(t),

with *X*(0) = *X*_0_.

Next, by applying the Ito's formula to *X* ^2^, we obtain

(31) dX2(t)={(2r−θTθ)X2(t)+2(k+θTqηf(t))L(t)X(t)+θTθγ2−θT(θ+qη)f(t)L(t)γ+(qTqη2+14θTθ+θTqη)f2(t)L2(t)}dt−2X(t)(θT(X(t)−γ+12f(t)L(t))+qTηL(t)f(t))dw(t),

with *X* ^2^(0) = *X*_0_^2^.

In addition, according to (2), we have *EL*(*t*) = *L*_0_ *e* ^*κt*^, *EL* ^2^(*t*) = *L*_0_^2^ *e* ^(2*κ*+*η* ^2^)*t*^.

So, by taking expectations on both previous stochastic differential equations, we obtain that functions *m*_1_(*t*) = *EX*(*t*) and *m*_2_(*t*) = *EX* ^2^(*t*) satisfy the linear ordinary differential equations

(32) m˙1(t)=(r−θTθ)m1(t)+kL0eκt    −θTθ(12f(t)L0eκt−γ),m1(0)=X0,m˙2(t)=(2r−θTθ)m2(t)+2(k+θTqηf(t))L0eκtm1(t)    +θTθγ2−θT(θ+2qη)f(t)L0eκtγ    +(qTqη2+14θTθ+θTqη)f2(t)L02e(2κ+η2)t,m2(0)=X02.

By solving (32), we can express *m*_1_(*T*) and *m*_2_(*T*) as explicit functions of *γ*

(33) $$\begin{array}{c} m_{1}\left(T\right)=\alpha_{T}X_{0}+\frac{\theta^{T}\theta }{r-\theta^{T}\theta }\left(\alpha_{T}-1\right)\gamma +\alpha_{T}\beta_{T}, \end{array}$$

with

(34) αt=e(r−θTθ)t,βt=L0∫0t(k−12θTθf(s))e(κ−r+θTθ)sds,m2(T)=αTerTX02+ϕγ2+φγ+ε,

and with

(35) ϕ=−θTθ2r−θTθ(1−αTerT),φ=αTerT{θTθr−θTθ∫0Tτt1λt(1−αt−1)dt−∫0Tτt2λtαtdt},ε=αTerT{X0∫0Tτt1λtdt+∫0Tτt1λtβtdt+∫0Tτt3αtλtdt},τt1=2(k+θTqηf(t)),τt2=θT(θ+2qη)f(t)L0,τt3=(qTqη2+14θTθ+θTqη)f2(t)L02e(κ+η2)t.

By using (33) and (34), we can thus give an explicit expression for problem (11), as a one parameter family in *γ*

(36) J¯(Λ∗(·),γ)=EX2(T)−2γEX(T)+2γK−K2=m2(T)−2γm1(T)+2γK−K2=αTerTX02+ϕγ2−2θTθr−θTθ(αT−1)γ2 −(2αTX0+2αTβT−2K−φ)γ+ε−K2;

that is, to say,

(37) min⁡ Λ(·)J¯(Λ(·),γ)=αTerTX02+ϕγ2−2θTθr−θTθ(αT−1)γ2 −(2αTX0+2αTβT−2K−φ)γ+ε−K2.

It then follows, using Remark 2, that minimum Var⁡*X*(*T*) is achieved for

(38) γ∗=2αTX0+2αTβT−2K−φ2ϕ−(4θTθ/(r−θTθ))(αT−1),

(39) min⁡ Var⁡X(T)=min⁡ Λ(·)J¯(Λ(·),γ∗)=(2αTX0+2αTβT−2K−φ)24((2θTθ/(r−θTθ))(αT−1)−ϕ) +αTerTX02−K2+ε.

**Proposition 7**

The efficient strategy of the portfolio selection problem (9) corresponding to the expected terminal wealth *EX*(*T*) = *K*, as a function of time *t*, wealth *X*(·), and γ* is given by

(40) Λ∗(t)=−Σ−1(b−r1)(X(t)−γ∗+12f(t)L(t)) −ησ−Tqf(t)L(t).

Moreover, the efficient frontier is

(41) Var⁡X(T)=(2αTX0+2αTβT−2EX(T)−φ)24((2θTθ/(r−θTθ  ))(αT−1)−ϕ) +αTerTX02−EX2(T)+ε.

**Remark 8**

Expression (41) shows the familiar quadratic relation between the wealth and its variance. The minimum possible variance is attained when

(42) $$\begin{array}{c} EX\left(T\right)=\frac{2\alpha_{T}X_{0}+2\alpha_{T}\beta_{T}-\varphi }{2+2\left(\left(2\theta^{T}\theta /\left(r-\theta^{T}\theta \right)\right)\left(\alpha_{T}-1\right)-\phi \right)}. \end{array}$$

**Remark 9**

Consider two wealth levels $\overset{-}{K}$ and $\tilde{K}\;\;\left(\in \left[X_{0}e^{rT}+\int_{0}^{T}kL(t)e^{r(T-t)}dt,+\infty \right),L(0)=L_{0}\right)$ and their corresponding efficient portfolio $\overset{-}{\Lambda }\left(\cdot \right)$ and $\tilde{\Lambda }\left(\cdot \right)$ given by the explicit formula (40). Then, a portfolio Λ*(·) is efficient if and only if $\Lambda^{*}\left(\cdot \right)=\lambda \overset{-}{\Lambda }\left(\cdot \right)+\left(1-\lambda \right)\tilde{\Lambda }\left(\cdot \right)$ for some *λ* ∈ *R* and corresponding to the wealth $K=\lambda \overset{-}{K}+\left(1-\lambda \right)\tilde{K}$ .

**Proof**

Suppose $\overset{-}{\Lambda }\left(\cdot \right)$ and $\tilde{\Lambda }\left(\cdot \right)$ are efficient and hence satisfy (40) corresponding to $\overset{-}{K}$ , $\tilde{K}$ , ${\overset{-}{\gamma }}^{*}$ , and ${\tilde{\gamma }}^{*}$ , respectively, and consider $\Lambda^{*}\left(\cdot \right)=\lambda \overset{-}{\Lambda }\left(\cdot \right)+\left(1-\lambda \right)\tilde{\Lambda }\left(\cdot \right)$ , with the corresponding γ*. By rearranging terms, Λ*(·) can be expressed in the form $\Lambda^{*}\left(\cdot \right)=\lambda \overset{-}{\Lambda }\left(\cdot \right)+\left(1-\lambda \right)\tilde{\Lambda }\left(\cdot \right)$ corresponding to $K=\lambda \overset{-}{K}+\left(1-\lambda \right)\tilde{K}$ and $\gamma^{*}=\lambda \overset{-}{\gamma }+\left(1-\lambda \right)\tilde{\gamma }$ and is therefore efficient. Conversely, suppose that Λ*(·) is efficient; that is, it satisfies (40) and corresponds to some *K*. Decompose *K* into $K=\lambda \overset{-}{K}+\left(1-\lambda \right)\tilde{K}$ for an appropriate *λ* ∈ *R*. Then, by using (38), write $\gamma^{*}=\lambda \overset{-}{\gamma }+\left(1-\lambda \right)\tilde{\gamma }$ and substitute back into (40) to arrive at the decomposition $\Lambda^{*}\left(\cdot \right)=\lambda \overset{-}{\Lambda }\left(\cdot \right)+\left(1-\lambda \right)\tilde{\Lambda }\left(\cdot \right)$ .

## 5. Conclusions

Defined-contribution pension funds play a crucial role in the social pension systems. The utility function is usually assumed to be a continuous, increasing, and strictly concave function such as a power, logarithm, exponential, or quadratic function. The risk and return relationship is implicit in the utility function approach and cannot be disentangled at the level of optimal strategies. In addition, the optimal asset allocation for a pension fund involves quite a long period, generally from 20 to 40 years, so it follows that it becomes crucial to take into account the salary risk. So this paper studies the optimal investment portfolio selection of DC pension funds with stochastic salary under the mean-variance model. We construct a special Riccati equation as a continuous (actually a viscosity) solution to the HJB equation and obtain an explicit closed form solution for the optimal investment portfolio as well as the efficient frontier.

In the future research, we will continue to concentrate on continuous-time portfolio selection problem under the mean-variance model. It would be interesting to extend our analysis to those more generalized situation, such as assuming the risky asset to follow constant elasticity of variance (CEV) models and introducing different stochastic interest rate model under the research framework. It is noteworthy that the optimal solver with the generalized situation is very difficult.

## References

[1] Boulier J-F, Huang S, Taillard G (2001). Optimal management under stochastic interest rates: the case of a protected defined contribution pension fund. *Insurance: Mathematics and Economics*.

[2] Deelstra G, Grasselli M, Koehl P-F (2003). Optimal investment strategies in the presence of a minimum guarantee. *Insurance: Mathematics and Economics*.

[3] Cairns AJG, Blake D, Dowd K (2006). Stochastic lifestyling: optimal dynamic asset allocation for defined contribution pension plans. *Journal of Economic Dynamics and Control*.

[4] Xiao J, Zhai H, Qin C (2007). The constant elasticity of variance (CEV) model and the Legendre transform-dual solution for annuity contracts. *Insurance: Mathematics and Economics*.

[5] Battocchio P, Menoncin F (2004). Optimal pension management in a stochastic framework. *Insurance: Mathematics and Economics*.

[6] Gao J (2009). Optimal portfolios for DC pension plans under a CEV model. *Insurance: Mathematics and Economics*.

[7] Gao J (2009). Optimal investment strategy for annuity contracts under the constant elasticity of variance (CEV) model. *Insurance: Mathematics and Economics*.

[8] Zhou XY, Li D (2000). Continuous-time mean-variance portfolio selection: a stochastic LQ framework. *Applied Mathematics and Optimization*.

[9] Markowitz HM (1952). Portfolio selection. *Journal of Finance*.

[10] Markowitz HM (1959). *Portfolio Selection: Efficient Diversification of Investment*.

[11] Campbell JY, Lo AW, MacKinlay AC (1997). *The Econometrics of Financial Markets*.

[12] Li D, Ng W-L (2000). Optimal dynamic portfolio selection: multiperiod mean-variance formulation. *Mathematical Finance*.

[13] Hakansson NH (1971). Capital growth and the mean-variance approach to portfolio selection. *Journal of Financial and Quantitative Analysis*.

[14] Merton RC (1972). An analytic derivation of the efficient portfolio frontier. *Journal of Financial and Economics Analysis*.

[15] Cox JC, Huang C-F (1989). Optimal consumption and portfolio policies when asset prices follow a diffusion process. *Journal of Economic Theory*.

[16] Grossman SJ, Zhou Z (1996). Equilibrium analysis of portfolio insurance. *Journal of Finance*.

[17] Merton RC (1971). Optimum consumption and portfolio rules in a continuous-time model. *Journal of Economic Theory*.

[18] Li X, Zhou XY, Lim AEB (2002). Dynamic mean-variance portfolio selection with non-shorting constraints. *SIAM Journal on Control and Optimization*.

[19] Chiu MC, Li D (2006). Asset and liability management under a continuous-time mean-variance optimization framework. *Insurance: Mathematics and Economics*.

[20] Xie S, Li Z, Wang S (2008). Continuous-time portfolio selection with liability: mean-variance model and stochastic LQ approach. *Insurance: Mathematics and Economics*.

[21] Fu C, Lari-Lavassani A, Li X (2010). Dynamic mean-variance portfolio selection with borrowing constraint. *European Journal of Operational Research*.

[22] Kramkov D, Schachermayer W (1999). The asymptotic elasticity of utility functions and optimal investment in incomplete markets. *The Annals of Applied Probability*.

[23] Jonsson M, Sircar R, Bourliux A, Gander M, Sabidussi G (2002). Optimal investment problems and volatility homogenization approximations. *Modern Methods in Scientific Computing and Appllications*.

